# Natural Bioactive Compounds and Human Health

**DOI:** 10.3390/molecules29143372

**Published:** 2024-07-18

**Authors:** Arunaksharan Narayanankutty, Ademola C. Famurewa, Eliza Oprea

**Affiliations:** 1 Division of Cell and Molecular Biology, PG and Research Department of Zoology, St. Joseph’s College (Autonomous), Devagiri, Kozhikode 673008, Kerala, India; 2 Department of Medical Biochemistry, Faculty of Basic Medical Sciences, College of Medicine, Alex Ekwueme Federal University, Ndufu-Alike lkwo, P.M.B. 1010, Abakaliki 482131, Nigeria; ademola.famurewa@funai.edu.ng; 3 Department of Botany and Microbiology, Faculty of Biology, University of Bucharest, 1-3 Portocalelor Street, 060101 Bucharest, Romania; eliza.oprea@g.unibuc.ro

## 1. Introduction

Natural bioactive compounds encompass a vast array of molecules derived from plants, fungi, marine organisms, and other natural sources [1]. Their biological activities range from antioxidant and anti-inflammatory properties to antimicrobial and anticancer effects, reflecting their potential as therapeutic agents and functional ingredients in healthcare and nutrition [2]. In recent decades, the exploration of natural bioactive compounds has emerged as a cornerstone in biomedical research, driven by a growing appreciation for their potential therapeutic benefits and contributions to human health. These compounds have garnered considerable attention not only for their efficacy but also for their relatively low toxicity profiles compared to their synthetic counterparts [3]. Their natural origins often align with traditional medicinal practices, validating centuries-old wisdom through modern scientific validation.

One of the defining characteristics of natural bioactive compounds is their structural diversity and complex mechanisms of action [4]. From polyphenols and alkaloids to terpenoids and carotenoids, each class exhibits unique chemical structures that dictate their interactions with biological systems. This diversity enables them to target specific pathways implicated in disease processes, offering tailored therapeutic strategies. Moreover, the synergy observed within natural extracts—where multiple bioactive compounds act collaboratively—enhances their overall efficacy and broadens their therapeutic potential. Understanding these interactions and harnessing them for therapeutic purposes represents a promising frontier in drug discovery and development [5].

Technological advancements in extraction and characterization techniques have significantly contributed to unlocking the full potential of natural bioactive compounds [6]. Ultrasonic-assisted extraction, supercritical fluid extraction, and chromatographic methods have revolutionized the isolation and purification of these compounds, enabling researchers to obtain high-purity extracts with optimized bioactivity [7]. Characterization techniques such as mass spectrometry, nuclear magnetic resonance (NMR) spectroscopy, and high-performance liquid chromatography (HPLC) provide detailed insights into the chemical composition and structural elucidation of bioactive compounds. These analytical tools are crucial for identifying novel compounds, elucidating their mechanisms of action, and ensuring consistency in quality and efficacy [8,9].

The therapeutic applications of natural bioactive compounds span a wide spectrum of health conditions, from chronic diseases to metabolic disorders and infectious diseases. Their antioxidant properties mitigate oxidative stress, a hallmark of aging and various chronic illnesses, while their anti-inflammatory effects modulate immune responses and reduce inflammatory markers implicated in diseases like arthritis, cardiovascular diseases, and neurodegenerative disorders [2]. Furthermore, their antimicrobial activities address the global challenge of antimicrobial resistance, offering alternative treatment options against drug-resistant pathogens [10]. In cancer therapy, bioactive compounds exhibit promising anticancer activities by targeting tumor cell proliferation, angiogenesis, and metastasis pathways, often with fewer side effects compared to conventional chemotherapy agents [11]. As research into natural bioactive compounds progresses, several emerging trends and future directions are shaping the field. These include exploring synergistic interactions between bioactive compounds and conventional therapies, developing nanoformulations to enhance bioavailability and targeted delivery, and integrating computational approaches to accelerate drug discovery and optimize molecular design [12]. Moreover, the incorporation of natural compounds into functional foods, dietary supplements, and cosmeceuticals underscores their role in preventive healthcare and wellness promotion. Regulatory frameworks and quality standards are also evolving to ensure the safety, efficacy, and sustainability of natural products, facilitating their translation from bench to bedside.

## 2. An Overview of the Contributions

*Artemisia nilagirica*, a plant native to the Western Ghats of India, showcases a rich phytochemical profile with potent antioxidant, anti-inflammatory, and anticancer properties (Albaqami et al., 2022) (Contribution 1).

*Rosa damascena* callus extracts, as studied by Darwish et al. (2022) (Contribution 3), offer insights into novel approaches in cancer therapy. Enhanced by vitamin-induced media, these extracts exhibit potent anti-proliferative effects against colorectal cancer cells, suggesting their role as adjuvant therapies in oncology.

*Polygonum cuspidatum*, optimized through ultrasonic-assisted extraction, as evaluated by Fletes-Vargas et al. (2023) (Contribution 4), demonstrates exceptional antioxidant capacity and cytotoxic activity against cancer cells. Such findings advocate for its integration into functional foods and pharmaceutical formulations targeting oxidative stress-related diseases.

The neuropharmacological effects of neophytadiene, as investigated by Gonzalez-Rivera et al. (2023) (Contribution 5), highlight its potential as an anxiolytic and anticonvulsant agent, shedding light on its mechanisms through GABAergic modulation, thus offering new avenues in mental health treatments.

The chemical composition evaluation of *Lonicera caerulea* varieties, explored by Gorzelany et al. (2023) (Contribution 6), unveils their rich antioxidant profiles and varying phenolic content, illustrating their potential for nutraceutical applications and dietary supplements.

Maslinic acid, reviewed comprehensively by He et al. (2022) (Contribution 7), emerges as a versatile compound with anti-inflammatory, antioxidant, and organ-protective properties, suggesting its therapeutic promise across diverse organ diseases.

Further, the synthetic derivatives of ganoderic acid A, as studied by Jia et al. (2023) (Contribution 8), demonstrate enhanced anticancer activities through modulation of the p53-MDM2 pathway, presenting a novel strategy in cancer therapeutics.

*Opuntia ficus-indica* seed oil, investigated by Alqurashi et al. (2022) (Contribution 2), emerges as another promising natural compound. This oil originates from Saudi Arabia and exhibits robust antioxidant properties as well as significant antiviral and antibacterial activities.

Kuttithodi et al. (2022) (Contribution 11) explore the antioxidant, antimicrobial, cytotoxicity, and larvicidal activities of synthetic bis-chalcones, highlighting their potential for further investigation in vivo.

Li et al. (2022) (Contribution 12) provide a comprehensive review of the application of metabolomics in fungal research, elucidating its role in identifying metabolites, understanding stress responses, and enhancing metabolic engineering in fungi.

Liu et al. (2023) (Contribution 13) investigate *Saussurea pulchella* for its comprehensive phytochemical composition and anti-ulcerative colitis effects, underscoring its potential as a natural treatment option for inflammatory bowel diseases.

Łysakowska et al. (2023) (Contribution 14) review medicinal mushrooms’ bioactive components and nutritional value, emphasizing their potential in functional food production due to their diverse health benefits.

Machado et al. (2022) (Contribution 15) discuss the improved solubility and permeability of psoralens from *Brosimum gaudichaudii* plant extract upon complexation with hydroxypropyl-β-cyclodextrin, highlighting advancements in drug formulation for enhanced bioavailability.

Menon et al. (2022) (Contribution 16) evaluate *Oroxylum indicum* root bark extract for its inhibitory effects on solid and ascites tumors, and its preventive potential against skin papilloma formation, revealing its promising anticancer properties.

Narayanankutty et al. (2022) (Contribution 17) investigated the chemical composition and biological activities of essential oils extracted from *Citrus limetta* peel waste, demonstrating their antioxidant, antibacterial, and anticancer properties, particularly rich in D-Limonene.

Nikiema et al. (2024) (Contribution 18) conducted a systematic review of immunomodulatory compounds isolated from African medicinal plants, highlighting their potential in managing inflammatory diseases and enhancing vaccine immunogenicity.

Paes et al. (2023) (Contribution 19) review phytocompounds from Amazonian plant species with potential nephroprotective effects against acute kidney injury, emphasizing the need for further pharmacological validation of these natural remedies.

Bioavailability and bioaccessibility of nutrients remain pivotal in nutritional science. Pasidi and Vareltzis (2024) (Contribution 20) explore the gastric pH effects on vitamin D3 bioaccessibility from supplements and foods. Their findings underscore the higher bioaccessibility of vitamin D3 from foods compared to supplements, influenced significantly by gastric pH variations. This study elucidates critical factors in enhancing nutrient absorption and informs strategies for dietary interventions.

Rajabi et al. (2023) (Contribution 21) review the role of long non-coding RNAs (LncRNAs) as targets for phytochemicals in inhibiting cancer metastasis, offering promising avenues for novel therapeutic developments.

Sidhic et al. (2023) (Contribution 24) investigate the phytochemical composition and biological activities of *Humboldtia sanjappae*, an endemic medicinal plant from the Western Ghats. Their findings reveal significant antioxidant, anti-inflammatory, and antibacterial properties, positioning H. sanjappae as a valuable resource for pharmaceutical and nutraceutical applications, particularly in combating oxidative stress and inflammatory disorders.

Sosa et al. (2023) (Contribution 25) explore the therapeutic applications of essential oils derived from native plants in treating cutaneous candidiasis and dermal inflammation. Their research underscores the efficacy of essential oils from *Bursera graveolens, Dacryodes peruviana*, *Mespilodaphne quixos*, and *Melaleuca armillaris* in combating fungal infections and reducing inflammatory responses, highlighting their potential as natural alternatives in dermatological therapies.

Shahid et al. (2023) (Contribution 23) contribute by examining the proximate composition and nutritional values of selected wild plants from the United Arab Emirates. Their study underscores the nutritional richness of plants such as *Chenopodium murale, Dipterygium glaucum*, and *Tribulus pentandrus*, emphasizing their potential as sustainable sources of essential nutrients and bioactive compounds in arid ecosystems.

Stepanova et al. (2022) (Contribution 26) discuss the collection of hairy roots as a basis for fundamental and applied research. Their review highlights the versatility of hairy root cultures in synthesizing bioactive compounds and underscores their potential in pharmaceutical and agricultural industries, offering sustainable solutions for producing plant-derived medicines and supplements.

Tom et al. (2023) (Contribution 28) present synthesized bis-chalcones and their cytoprotective and anti-inflammatory effects. Their study demonstrates the therapeutic potential of bis-chalcones in protecting intestinal epithelial cells from oxidative stress and modulating inflammatory responses, suggesting these compounds as promising candidates for developing novel anti-inflammatory agents.

Xu et al. (2023) (Contribution 32) investigate the protective effects of Wendan Decoction on endothelial cell injury induced by hyperlipidemia. Their study identifies active ingredients such as trigonelline, liquiritin, and quercetin, highlighting the therapeutic potential of traditional Chinese herbal medicine in cardiovascular health, underscoring the significance of integrating ancient wisdom with modern scientific methodologies.

Zhang et al. (2023) (Contribution 33) explore the chemical fingerprinting and bioactivity of *Polygonatum cyrtonema* before and after processing with black bean juice. Their findings reveal enhanced antioxidant and anti-diabetic activities post-processing, shedding light on traditional processing techniques to optimize the therapeutic efficacy of Chinese herbal medicines.

Wei and Zhang (2024) (Contribution 31) review the anti-angiogenic properties of flavonoids in cancer therapy. Their comprehensive analysis elucidates the mechanisms by which plant-derived flavonoids inhibit angiogenesis, highlighting their potential as natural anticancer agents.

Teixeira et al. (2023) (Contribution 27) contribute insights into Brazilian green propolis extraction methods and their antioxidant profiles. Their investigation underscores the impact of ultrasound-assisted and high-pressure extraction on flavonoid and phenolic content, highlighting optimal conditions for maximizing antioxidant activity in propolis extracts.

Jiménez-Ferrer et al. (2022) (Contribution 9) explore the anti-inflammatory and immunomodulatory properties of *Agave angustifolia*. Their investigation highlights the acetonic fraction’s efficacy in reducing articular edema, spleen index, and pain in a mouse model, with GC-MS analysis identifying key fatty acid derivatives responsible for these effects.

Kietrungruang et al. (2023) (Contribution 10) evaluate propolis-loaded niosomes (Nio-EEP) against *Cryptococcus neoformans*. Their study demonstrates the niosomes’ significant impact on reducing PLB1 production, biofilm formation, and intracellular replication of the fungus, proposing Nio-EEP as a potential therapeutic strategy for pulmonary cryptococcosis.

Sabira et al. (2022) (Contribution 22) analyze the defensive gland extract of *Luprops tristis*, revealing compounds with significant antibacterial, antioxidant, and cytotoxic activities. Their study highlights the extract’s potential therapeutic applications despite the beetle’s harmful reputation.

Vij et al. (2023) (Contribution 29) review the bioactive compounds in *Caesalpinia sappan*, emphasizing their antioxidant, anti-inflammatory, and anticancer properties. Their review underscores the potential of sappan wood in traditional medicine and drug development, focusing on compounds such as brazilin and brazilein.

Wang et al. (2023) (Contribution 30) assess the antihypertensive effects of acetylcholine (ACh) and γ-aminobutyric acid (GABA) in eggplant. Their study indicates that ACh is the primary compound responsible for blood pressure reduction, suggesting it could be a more effective functional food constituent for hypertension management than GABA.

Ziemlewska et al. (2022) (Contribution 34) investigate the cosmetic properties of mushroom extracts from Reishi, Maitake, and Lion’s Mane. Their study highlights the extracts’ antioxidant properties, skin hydration benefits, and reduced skin irritation, proposing their potential in natural cosmetic formulations.
